# Synthesis, crystal structure and computational studies of a new Schiff base compound: (*E*)-4-bromo-2-eth­oxy-6-{[(2-meth­oxy­phen­yl)imino]meth­yl}phenol

**DOI:** 10.1107/S2056989018002062

**Published:** 2018-02-07

**Authors:** Arzu Özek Yıldırım, Murat Gülsu, Çiğdem Albayrak Kaştaş

**Affiliations:** a Department of Physics, Faculty of Arts and Sciences, Giresun University, Turkey; b Department of Chemistry, Faculty of Arts and Sciences, Sinop University, Turkey

**Keywords:** Schiff bas, crystal structure, DFT, 5-bromo-3-eth­oxy-2-hy­droxy­benzaldehyde, 2-meth­oxy­aniline

## Abstract

The title compound, C_16_H_16_BrNO_3_, which shows enol–imine tautomerism, crystallizes in the monoclinic *P*2_1_/*c* space group. All non-H atoms of the mol­ecule are nearly coplanar, with a maximum deviation of 0.274 (3) Å. In the crystal, mol­ecules are held together by weak C—H⋯O, π–π and C—H⋯π inter­actions. The *E*/*Z* isomerism and enol/keto tautomerism energy barriers of the compound have been calculated by relaxed potential energy surface scan calculations with DFT methods. To observe the changes in the aromatic ring, HOMA aromaticity indexes were calculated during the scan process. Total energy and HOMA change curves were obtained to visualize results of the scan calculations.

## Abstract 2

The title compound has enol–imine tautomeric form. *E*/*Z* isomerism and enol/keto tautomerism energy barriers have been calculated by relaxed potential energy surface scan calculations with DFT methods.

## Chemical context   

The synthesis and chemistry of Schiff bases have received considerable attention over the last several decades, primarily owing to their remarkable potential pharmacological (Hu *et al.*, 2012 ▸), anti-tumor (Kamel *et al.*, 2010 ▸) and biological properties (Lozier *et al.*, 1975 ▸). Furthermore, Schiff bases can display photo-chromic and thermo-chromic effect (Hadjoudis & Mavridis, 2004 ▸). These effects depend on the prototropic tautomerism and mol­ecular planarity in Schiff bases (Moustakali-Mavridis *et al.*, 1978 ▸; Hadjoudis *et al.*, 1987 ▸). Prototropic tautomerism emerges from the intra­molecular H-atom transfer between an enol–imine (Özdemir Tarı *et al.*, 2016 ▸) and a keto–amine tautomer (Özek *et al.*, 2006 ▸). The present work is part of our ongoing studies on Schiff bases (Özek Yıldırım *et al.*, 2016 ▸, 2017 ▸; Albayrak *et al.*, 2012 ▸). We report herein the synthesis, crystal structure and computational studies of the title compound, (*E*)-4-bromo-2-eth­oxy-6-{[(2-meth­oxy­phen­yl)imino]­meth­yl}phenol, obtained from the condensation of 5-bromo-3-eth­oxy-2-hy­droxy­benzaldehyde with 2-meth­oxy­aniline.
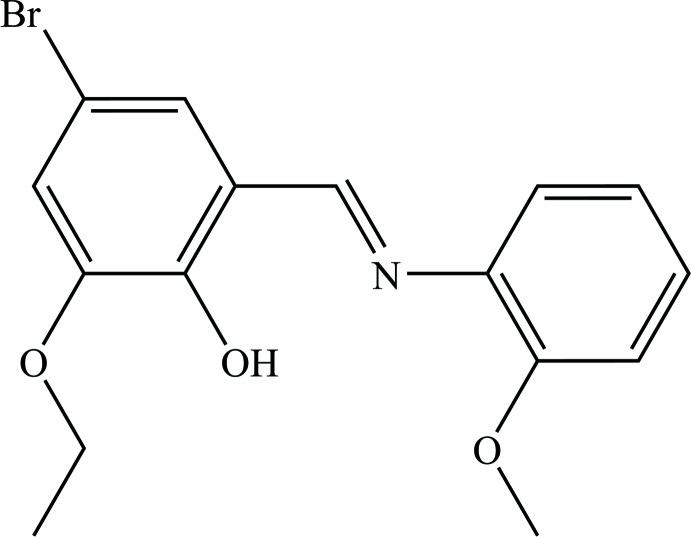


## Structural commentary   

**Figure 1:**
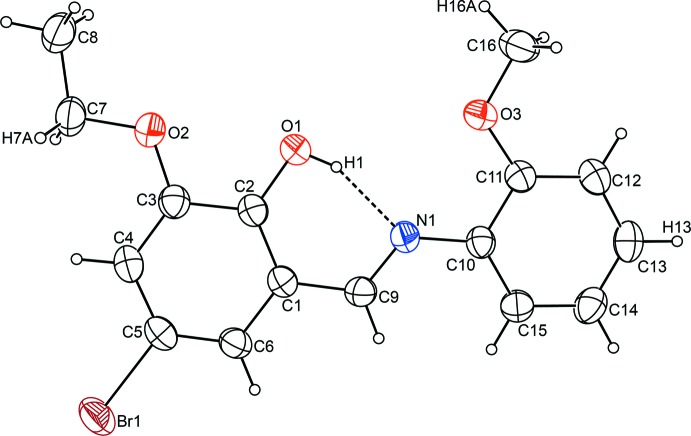
The mol­ecular structure of the title compound, with atom labels and 50% probability displacement ellipsoids for non-H atoms. The dashed line indicates the intra­molecular hydrogen bond.

Fig. 1 ▸ represents the mol­ecular structure of the title compound. All non-H atoms lie in the plane formed by the aromatic rings with a maximum deviation of 0.274 (3) Å. The dihedral angle between the aromatic rings C1–C6 and C10–C15 is 2.25 (13)°. In the chelate moiety, which comprises atoms C1, C2, O1, H1, N1 and C9, C9=N1 [1.281 (3)] is a typical double bond while C2—O1 [1.333 (3)] is a typical single bond; these are similar to those in related structures (Petek *et al.*, 2010 ▸; Gül *et al.*, 2007 ▸). The harmonic oscillator model of aromaticity (HOMA; Kruszewski & Krygowski, 1972 ▸) values were calculated [0.88 for C1–C6 and 0.98 for the C10–C15 ring] to observe the effect of substituent groups on the rings. There are no significant deformations of the rings when compared to those in (*E*)-2-eth­oxy-6-[(2-meth­oxy­phenyl­imino)­meth­yl]phenol (Petek *et al.*, 2010 ▸). The chelate moiety forms an *S*(6) graph-set motif through a strong intra­molecular O1—H1⋯N1 hydrogen bond (Table 1 ▸).

## Supra­molecular features   

**Figure 2:**
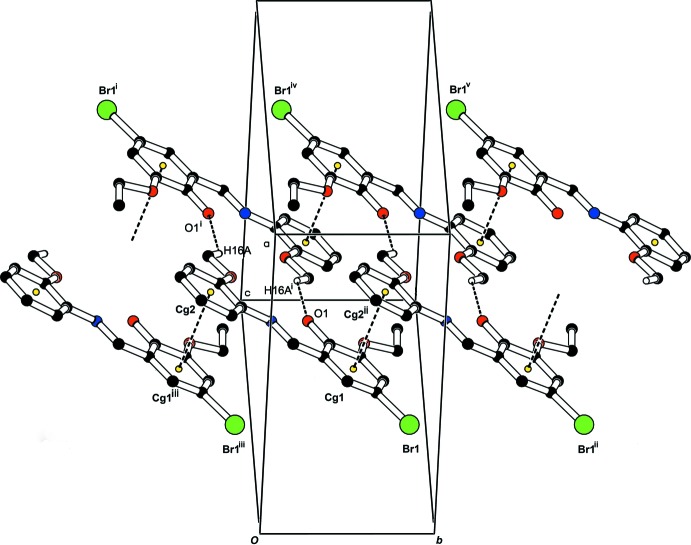
View of the inversion dimers, which are connected by π–π inter­actions, propagating along the *c-*axis direction. [Symmetry codes: (i) −*x* + 1, −*y*, −*z* + 1; (ii) *x*, *y* + 1, *z*; (iii) *x*, *y* − 1, *z*; (iv) −*x* + 1, −*y* + 1, −*z* + 1; (v) −*x* + 1, −*y* + 2, −*z* + 1.]

**Figure 3:**
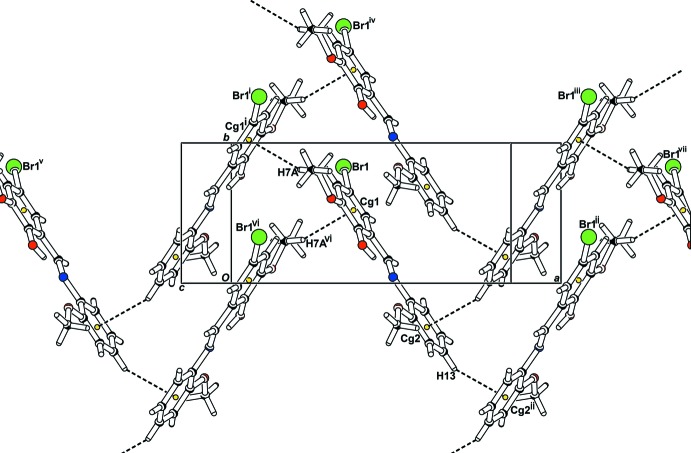
The packing, viewed down the *c* axis, showing mol­ecules connected by C—H⋯π inter­actions [Symmetry codes: (i) −*x* + 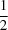, *y* + 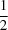, −*z* + 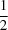; (ii) −*x* + 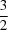, *y* − 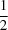, −*z* + 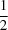; (iii) −*x* + 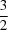, *y* + 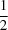, −*z* + 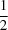; (iv) *x*, *y* + 1, *z*; (v) *x* − 1, *y*, *z*; (vi) −*x* + 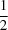, *y* − 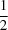, −*z* + 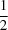; (vii) *x* + 1, *y*, *z*.]

In the crystal, inversion dimers with an 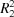 motif are generated by the weak C16—H16*A*⋯O1(−*x* + 1, −*y*, −*z* + 1) hydrogen bonds (Table 1 ▸). As shown in Fig. 2 ▸, these dimers are connected to each other by π–π inter­actions [*Cg*1⋯*Cg*2(*x*, *y* + 1, *z*) = 3.6237 (16) Å; *Cg*1 and *Cg*2 are the centroids of the C1–C6 and C10–C15 rings, respectively]. C—H⋯π inter­actions (Table 1 ▸) generate zigzag chains along the [100] direction as shown in Fig. 3 ▸.

## Computational Studies   

**Figure 4:**
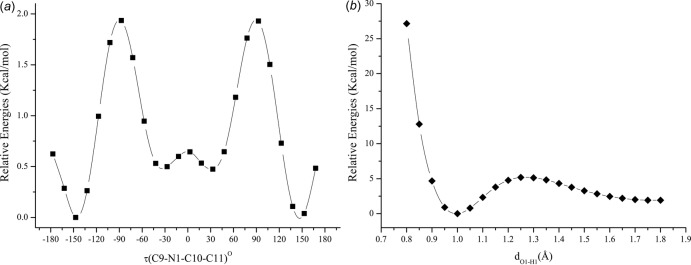
The potential energy curves for the torsional scan (*a*) and the O—H bond scan (*b*). Relative energies are calculated with respect to the global minimum of each curve.

**Figure 5:**
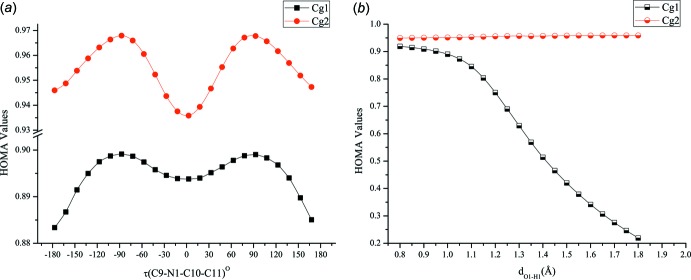
Graphics showing the variation of HOMA values with scan coordinate.

Relaxed potential energy surface scan calculations were performed using the DFT/B3LYP/6-311G++(d,p) method with *Gaussian 09W* software (Frisch *et al.*, 2009 ▸) to investigate the connection between the mol­ecular conformation and physical properties of a Schiff base. The results of a torsional angle scan and a proton-transfer scan on the O—H⋯N pathway are given in Fig. 4 ▸. The torsional barrier between the *E*/*Z* isomers was found to be 1.94 kcal mol^−1^ and the enol–keto tautomerism barrier was 1.92 kcal mol^−1^. The effects of the conformational changes on the aromatic ring can be visualized by calculating HOMA values during the scan calculations. Fig. 5 ▸ *a* shows that changes in the HOMA indices are very limited with an average fluctuation of 2%. As can be seen in Fig. 5 ▸ *b*, the aromaticity of the C1–C6 ring depends strongly on the prototropic tautomerism.

## Database survey   

A survey of the Cambridge Structural Database (CSD, Version 5.37, update May 2017; Groom *et al.*, 2016 ▸) for the (*E*)-4-bromo-2-eth­oxy-6-[(methyl­imino)­meth­yl]phenol unit of the title compound reveals five compounds, *viz*. OCOVEK (Kaştaş *et al.*, 2017*a* ▸), OCOVIO (Kaştaş *et al.*, 2017*b* ▸), OCOVOU (Kaştaş *et al.*, 2017*c* ▸), OCOVUA (Kaştaş *et al.*, 2017*d* ▸) and LUWZIO (Özek Yıldırım *et al.*, 2016 ▸). The mol­ecular structures of the latter two compounds are planar, in which they are similar to the title compound, while the others are not planar.

## Synthesis and crystallization   

The title compound was prepared by refluxing a mixture of a solution containing 5-bromo-3-eth­oxy-2-hy­droxy­benzaldehyde (0.5 g, 2 mmol) in 20 ml ethanol and a solution containing 2-meth­oxy­aniline (0.25 g, 2 mmol) in 20 ml ethanol. The reaction mixture was stirred for 1 h under reflux. Crystals suitable for X-ray analysis were obtained from an ethanol solution by slow evaporation (yield 70%).

## Refinement   

Crystal data, data collection and structure refinement details are summarized in Table 2 ▸. The hydroxyl atom H1 was refined freely. All the other H atoms were located geometrically and refined using a riding model with C—H = 0.93–0.97 Å *U*_iso_(H) = 1.2*U*_eq_(C).

## Supplementary Material

Crystal structure: contains datablock(s) I, global. DOI: 10.1107/S2056989018002062/xu5918sup1.cif

Structure factors: contains datablock(s) I. DOI: 10.1107/S2056989018002062/xu5918Isup2.hkl

Supporting information file. DOI: 10.1107/S2056989018002062/xu5918Isup3.cml

CCDC reference: 1457124

Additional supporting information:  crystallographic information; 3D view; checkCIF report

## Figures and Tables

**Table 1:** Hydrogen-bond geometry (Å, °) *Cg*1 and *Cg*2 are the centroids of the C1–C6 and C10–C15 rings, respectively.

*D*—H⋯*A*	*D*—H	H⋯*A*	*D*⋯*A*	*D*—H⋯*A*
O1—H1⋯N1	0.81 (5)	1.80 (5)	2.566 (3)	157 (5)
C16—H16*A*⋯O1^i^	0.96	2.55	3.293 (3)	135
C7—H7*A*⋯*Cg*1^ii^	0.97	2.80	3.662 (3)	149
C13—H13⋯*Cg*2^iii^	0.93	2.79	3.629 (3)	150

Symmetry codes: (i) 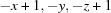; (ii) 
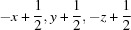
; (iii) 
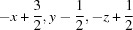
.

**Table 2:** Experimental details

Crystal data	
Chemical formula	C_16_H_16_BrNO_3_
*M*_r_	350.21
Crystal system, space group	Monoclinic, *P*2_1_/*n*
Temperature (K)	296
*a*, *b*, *c* (Å)	15.3405 (8), 6.5204 (2), 15.3612 (10)
β (°)	98.716 (5)
*V* (Å^3^)	1518.78 (14)
*Z*	4
Radiation type	Mo *K*α
μ (mm^−1^)	2.72
Crystal size (mm)	0.56 × 0.28 × 0.05

Data collection	
Diffractometer	Stoe IPDS 2
Absorption correction	Integration (*X-RED32*; Stoe & Cie, 2002 ▸)
*T*_min_, *T*_max_	0.437, 0.893
No. of measured, independent and observed [*I* > 2σ(*I*)] reflections	18156, 3491, 2754
*R*_int_	0.042
(sin θ/λ)_max_ (Å^−1^)	0.650

Refinement	
*R*[*F* ^2^ > 2σ(*F* ^2^)], *wR*(*F* ^2^), *S*	0.042, 0.091, 1.06
No. of reflections	3491
No. of parameters	194
H-atom treatment	H atoms treated by a mixture of independent and constrained refinement
Δρ_max_, Δρ_min_ (e Å^−3^)	0.28, −0.38

Computer programs: *X-AREA* and *X-RED32* (Stoe & Cie, 2002 ▸), *SHELXS97* (Sheldrick, 2015 ▸), *SHELXL2018* (Sheldrick, 2015 ▸), *ORTEP-3 for Windows* and *WinGX* (Farrugia, 2012 ▸) and *PLATON* (Spek, 2009 ▸).

## supplementary crystallographic information

### Crystal data

**Table:**

C_16_H_16_BrNO_3_	*F*(000) = 712
*M**_r_* = 350.21	*D*_x_ = 1.532 Mg m^−^^3^
Monoclinic, *P*2_1_/*n*	Mo *K*α radiation, λ = 0.71073 Å
*a* = 15.3405 (8) Å	Cell parameters from 3491 reflections
*b* = 6.5204 (2) Å	θ = 2.0–28.1°
*c* = 15.3612 (10) Å	µ = 2.72 mm^−^^1^
β = 98.716 (5)°	*T* = 296 K
*V* = 1518.78 (14) Å^3^	Prism, orange
*Z* = 4	0.56 × 0.28 × 0.05 mm

### Data collection

**Table:**

Stoe IPDS 2 diffractometer	3491 independent reflections
Radiation source: sealed X-ray tube, 12 x 0.4 mm long-fine focus	2754 reflections with *I* > 2σ(*I*)
Detector resolution: 6.67 pixels mm^-1^	*R*_int_ = 0.042
rotation method scans	θ_max_ = 27.5°, θ_min_ = 2.0°
Absorption correction: integration (X-RED32; Stoe & Cie, 2002)	*h* = −19→19
*T*_min_ = 0.437, *T*_max_ = 0.893	*k* = −8→8
18156 measured reflections	*l* = −19→19

### Refinement

**Table:**

Refinement on *F*^2^	0 restraints
Least-squares matrix: full	Hydrogen site location: mixed
*R*[*F*^2^ > 2σ(*F*^2^)] = 0.042	H atoms treated by a mixture of independent and constrained refinement
*wR*(*F*^2^) = 0.091	*w* = 1/[σ^2^(*F*_o_^2^) + (0.0398*P*)^2^ + 0.5265*P*] where *P* = (*F*_o_^2^ + 2*F*_c_^2^)/3
*S* = 1.06	(Δ/σ)_max_ = 0.001
3491 reflections	Δρ_max_ = 0.28 e Å^−^^3^
194 parameters	Δρ_min_ = −0.38 e Å^−^^3^

### Special details

**Table:**

Geometry. All esds (except the esd in the dihedral angle between two l.s. planes) are estimated using the full covariance matrix. The cell esds are taken into account individually in the estimation of esds in distances, angles and torsion angles; correlations between esds in cell parameters are only used when they are defined by crystal symmetry. An approximate (isotropic) treatment of cell esds is used for estimating esds involving l.s. planes.

### Fractional atomic coordinates and isotropic or equivalent isotropic displacement parameters (Å^2^)

**Table:**

	*x*	*y*	*z*	*U*_iso_*/*U*_eq_	
C1	0.44857 (16)	0.3446 (4)	0.18253 (15)	0.0403 (5)	
C2	0.42497 (17)	0.3902 (4)	0.26511 (16)	0.0417 (5)	
C3	0.37588 (17)	0.5714 (4)	0.27491 (17)	0.0440 (6)	
C4	0.35124 (17)	0.6978 (4)	0.20324 (18)	0.0469 (6)	
H4	0.318414	0.815670	0.208919	0.056*	
C5	0.37596 (17)	0.6473 (4)	0.12266 (17)	0.0456 (6)	
C6	0.42285 (18)	0.4758 (4)	0.11072 (16)	0.0457 (6)	
H6	0.437777	0.445619	0.055674	0.055*	
C7	0.3190 (2)	0.7984 (4)	0.3743 (2)	0.0574 (7)	
H7A	0.260045	0.809407	0.341291	0.069*	
H7B	0.354488	0.909669	0.356734	0.069*	
C8	0.3155 (2)	0.8097 (5)	0.4713 (2)	0.0640 (8)	
H8A	0.290087	0.938248	0.484727	0.077*	
H8B	0.280127	0.699194	0.487896	0.077*	
H8C	0.374171	0.799032	0.503274	0.077*	
C9	0.50092 (17)	0.1655 (4)	0.17069 (16)	0.0442 (5)	
H9	0.516428	0.138217	0.115620	0.053*	
C10	0.57777 (16)	−0.1337 (4)	0.22892 (16)	0.0406 (5)	
C11	0.59676 (16)	−0.2499 (4)	0.30608 (16)	0.0420 (5)	
C12	0.64681 (19)	−0.4279 (4)	0.30608 (19)	0.0513 (6)	
H12	0.659509	−0.505656	0.357194	0.062*	
C13	0.67759 (18)	−0.4892 (5)	0.2304 (2)	0.0550 (7)	
H13	0.711082	−0.608217	0.230857	0.066*	
C14	0.6594 (2)	−0.3766 (5)	0.1542 (2)	0.0552 (7)	
H14	0.680358	−0.419005	0.103432	0.066*	
C15	0.60944 (19)	−0.1994 (4)	0.15371 (18)	0.0508 (6)	
H15	0.596978	−0.123232	0.102130	0.061*	
C16	0.5727 (2)	−0.2971 (5)	0.45460 (19)	0.0622 (8)	
H16A	0.546330	−0.227372	0.499090	0.075*	
H16B	0.544000	−0.426840	0.441808	0.075*	
H16C	0.634215	−0.319190	0.475376	0.075*	
N1	0.52630 (14)	0.0438 (3)	0.23498 (13)	0.0423 (5)	
O1	0.44838 (15)	0.2719 (3)	0.33548 (12)	0.0536 (5)	
O2	0.35740 (14)	0.6041 (3)	0.35724 (12)	0.0546 (5)	
O3	0.56318 (14)	−0.1756 (3)	0.37679 (12)	0.0558 (5)	
Br1	0.34398 (2)	0.82939 (5)	0.02613 (2)	0.06603 (13)	
H1	0.479 (3)	0.186 (8)	0.316 (3)	0.119 (18)*	

### Atomic displacement parameters (Å^2^)

**Table:**

	*U*^11^	*U*^22^	*U*^33^	*U*^12^	*U*^13^	*U*^23^
C1	0.0405 (12)	0.0374 (12)	0.0424 (12)	−0.0003 (11)	0.0041 (10)	0.0031 (10)
C2	0.0433 (13)	0.0374 (13)	0.0443 (12)	0.0007 (10)	0.0064 (10)	0.0027 (10)
C3	0.0429 (14)	0.0415 (13)	0.0483 (13)	0.0002 (11)	0.0092 (11)	0.0009 (11)
C4	0.0441 (14)	0.0383 (14)	0.0583 (15)	0.0045 (11)	0.0081 (11)	0.0060 (11)
C5	0.0446 (14)	0.0415 (14)	0.0493 (13)	−0.0018 (11)	0.0021 (11)	0.0109 (11)
C6	0.0492 (14)	0.0476 (15)	0.0403 (12)	0.0025 (12)	0.0070 (11)	0.0067 (11)
C7	0.0640 (18)	0.0433 (16)	0.0674 (18)	0.0125 (13)	0.0184 (14)	0.0005 (13)
C8	0.074 (2)	0.0519 (17)	0.0698 (19)	0.0103 (15)	0.0230 (16)	−0.0074 (15)
C9	0.0514 (14)	0.0430 (13)	0.0387 (12)	−0.0004 (12)	0.0087 (10)	0.0010 (11)
C10	0.0404 (13)	0.0363 (13)	0.0458 (12)	−0.0001 (10)	0.0089 (10)	0.0016 (10)
C11	0.0392 (13)	0.0422 (13)	0.0450 (12)	−0.0001 (11)	0.0080 (10)	0.0031 (10)
C12	0.0491 (15)	0.0465 (15)	0.0576 (15)	0.0062 (12)	0.0061 (12)	0.0089 (13)
C13	0.0458 (15)	0.0433 (15)	0.0764 (19)	0.0074 (12)	0.0106 (14)	−0.0017 (14)
C14	0.0555 (17)	0.0534 (17)	0.0607 (16)	0.0041 (13)	0.0217 (13)	−0.0073 (13)
C15	0.0580 (16)	0.0498 (16)	0.0475 (14)	0.0037 (13)	0.0170 (12)	0.0037 (12)
C16	0.070 (2)	0.070 (2)	0.0473 (15)	0.0027 (16)	0.0107 (13)	0.0152 (14)
N1	0.0467 (12)	0.0375 (11)	0.0436 (10)	0.0035 (9)	0.0092 (9)	0.0030 (9)
O1	0.0730 (14)	0.0473 (11)	0.0425 (10)	0.0160 (10)	0.0152 (9)	0.0081 (8)
O2	0.0703 (13)	0.0445 (10)	0.0519 (10)	0.0137 (9)	0.0184 (9)	0.0025 (8)
O3	0.0709 (13)	0.0559 (11)	0.0434 (9)	0.0145 (10)	0.0176 (9)	0.0106 (9)
Br1	0.0763 (2)	0.05858 (19)	0.06202 (19)	0.01265 (16)	0.00668 (14)	0.02361 (15)

### Geometric parameters (Å, º)

**Table:**

C1—C2	1.403 (3)	C9—N1	1.281 (3)
C1—C6	1.405 (3)	C9—H9	0.9300
C1—C9	1.444 (4)	C10—C15	1.387 (4)
C2—O1	1.333 (3)	C10—C11	1.400 (3)
C2—C3	1.421 (4)	C10—N1	1.412 (3)
C3—O2	1.354 (3)	C11—O3	1.360 (3)
C3—C4	1.381 (4)	C11—C12	1.392 (4)
C4—C5	1.388 (4)	C12—C13	1.379 (4)
C4—H4	0.9300	C12—H12	0.9300
C5—C6	1.356 (4)	C13—C14	1.373 (4)
C5—Br1	1.905 (2)	C13—H13	0.9300
C6—H6	0.9300	C14—C15	1.386 (4)
C7—O2	1.438 (3)	C14—H14	0.9300
C7—C8	1.500 (4)	C15—H15	0.9300
C7—H7A	0.9700	C16—O3	1.423 (3)
C7—H7B	0.9700	C16—H16A	0.9600
C8—H8A	0.9600	C16—H16B	0.9600
C8—H8B	0.9600	C16—H16C	0.9600
C8—H8C	0.9600	O1—H1	0.81 (5)
			
C2—C1—C6	120.0 (2)	N1—C9—H9	119.5
C2—C1—C9	120.7 (2)	C1—C9—H9	119.5
C6—C1—C9	119.3 (2)	C15—C10—C11	118.9 (2)
O1—C2—C1	122.4 (2)	C15—C10—N1	125.3 (2)
O1—C2—C3	118.5 (2)	C11—C10—N1	115.8 (2)
C1—C2—C3	119.1 (2)	O3—C11—C12	125.0 (2)
O2—C3—C4	125.4 (2)	O3—C11—C10	115.3 (2)
O2—C3—C2	114.8 (2)	C12—C11—C10	119.7 (2)
C4—C3—C2	119.8 (2)	C13—C12—C11	120.1 (3)
C3—C4—C5	119.4 (2)	C13—C12—H12	120.0
C3—C4—H4	120.3	C11—C12—H12	120.0
C5—C4—H4	120.3	C14—C13—C12	120.8 (3)
C6—C5—C4	122.6 (2)	C14—C13—H13	119.6
C6—C5—Br1	119.2 (2)	C12—C13—H13	119.6
C4—C5—Br1	118.23 (19)	C13—C14—C15	119.5 (3)
C5—C6—C1	119.2 (2)	C13—C14—H14	120.3
C5—C6—H6	120.4	C15—C14—H14	120.3
C1—C6—H6	120.4	C14—C15—C10	121.1 (3)
O2—C7—C8	107.6 (2)	C14—C15—H15	119.5
O2—C7—H7A	110.2	C10—C15—H15	119.5
C8—C7—H7A	110.2	O3—C16—H16A	109.5
O2—C7—H7B	110.2	O3—C16—H16B	109.5
C8—C7—H7B	110.2	H16A—C16—H16B	109.5
H7A—C7—H7B	108.5	O3—C16—H16C	109.5
C7—C8—H8A	109.5	H16A—C16—H16C	109.5
C7—C8—H8B	109.5	H16B—C16—H16C	109.5
H8A—C8—H8B	109.5	C9—N1—C10	124.4 (2)
C7—C8—H8C	109.5	C2—O1—H1	102 (3)
H8A—C8—H8C	109.5	C3—O2—C7	117.3 (2)
H8B—C8—H8C	109.5	C11—O3—C16	118.0 (2)
N1—C9—C1	120.9 (2)		
			
C6—C1—C2—O1	−179.4 (2)	N1—C10—C11—O3	−0.2 (3)
C9—C1—C2—O1	−0.7 (4)	C15—C10—C11—C12	0.1 (4)
C6—C1—C2—C3	−0.7 (4)	N1—C10—C11—C12	179.7 (2)
C9—C1—C2—C3	177.9 (2)	O3—C11—C12—C13	180.0 (3)
O1—C2—C3—O2	−0.3 (4)	C10—C11—C12—C13	0.1 (4)
C1—C2—C3—O2	−179.0 (2)	C11—C12—C13—C14	−0.1 (4)
O1—C2—C3—C4	179.5 (2)	C12—C13—C14—C15	0.0 (5)
C1—C2—C3—C4	0.8 (4)	C13—C14—C15—C10	0.1 (5)
O2—C3—C4—C5	179.0 (2)	C11—C10—C15—C14	−0.2 (4)
C2—C3—C4—C5	−0.9 (4)	N1—C10—C15—C14	−179.8 (3)
C3—C4—C5—C6	0.9 (4)	C1—C9—N1—C10	−179.8 (2)
C3—C4—C5—Br1	−178.1 (2)	C15—C10—N1—C9	1.3 (4)
C4—C5—C6—C1	−0.7 (4)	C11—C10—N1—C9	−178.3 (2)
Br1—C5—C6—C1	178.21 (19)	C4—C3—O2—C7	−8.4 (4)
C2—C1—C6—C5	0.7 (4)	C2—C3—O2—C7	171.4 (2)
C9—C1—C6—C5	−178.0 (2)	C8—C7—O2—C3	−172.8 (2)
C2—C1—C9—N1	0.6 (4)	C12—C11—O3—C16	−6.0 (4)
C6—C1—C9—N1	179.3 (2)	C10—C11—O3—C16	173.9 (2)
C15—C10—C11—O3	−179.8 (2)		

### Hydrogen-bond geometry (Å, º)

Cg1 and Cg2 are the centroids of the C1–C6 and C10–C15 rings, respectively.

**Table:**

*D*—H···*A*	*D*—H	H···*A*	*D*···*A*	*D*—H···*A*
O1—H1···N1	0.81 (5)	1.80 (5)	2.566 (3)	157 (5)
C16—H16*A*···O1^i^	0.96	2.55	3.293 (3)	135
C7—H7*A*···*Cg*1^ii^	0.97	2.80	3.662 (3)	149
C13—H13···*Cg*2^iii^	0.93	2.79	3.629 (3)	150

Symmetry codes: (i) −*x*+1, −*y*, −*z*+1; (ii) −*x*+1/2, *y*+1/2, −*z*+1/2; (iii) −*x*+3/2, *y*−1/2, −*z*+1/2.
